# Embracing the Value of *Amanah*: A Guiding Principle for Medical School Academicians

**DOI:** 10.21315/mjms-12-2024-1003

**Published:** 2025-02-28

**Authors:** Kamarul Aryffin Baharuddin, Irfan Mohamad, Asrenee Ab Razak, Wan Mohd Nazaruddin Wan Hassan, Aziah Daud, Nik Ahmad Zuky Nik Lah, Syuhazlina Marini Awg Mat

**Affiliations:** School of Medical Sciences, Universiti Sains Malaysia, Health Campus, Kelantan, Malaysia

**Keywords:** academician, medical school, excellence, accountability, time management

## Abstract

Excellence in medical school demands a steadfast commitment to *amanah*—a trust upheld with integrity and accountability. This responsibility spans academics, student mentorship, research, and service, requiring academicians to balance their roles. A good understanding of curricular principles for undergraduate and postgraduate programmes, strengthened by robust quality assurance, serves as the cornerstone of academic excellence. The educational agenda, guided by the value-driven HEBAT framework and seamlessly integrated in diverse programmes, aims to boost student excellence. Related initiatives nurture intercultural competence and shape compassionate, resilient professionals equipped with the insight needed to navigate and excel in the complexities of modern medical practice.

Research clusters foster innovation and promote interdisciplinary collaboration. By balancing team dynamics and focused groups, they can enhance productivity and impact. Aligning community service with the Sustainable Development Goals requires strategies for properly documenting and measuring impact. A hallmark of academic excellence is time management which enables academicians to harmonise their responsibilities. The way forward for medical schools lies in enriching collaborations and international engagement. Together, these efforts affirm the value of *amanah* of academicians to drive excellence.

## Introduction

*Amanah* is an Arabic term that signifies trust, responsibility, and integrity. In an academic context, it refers to the ethical obligations and moral duties that scholars and educators must uphold. *Amanah* represents the capacity to meet the expectations of those who trust you and to integrate the necessary skills to fulfil your responsibilities, with a strong commitment to integrity and accountability (1).

In academia, *amanah* is a fundamental principle which guides personal and professional conduct (2). It sets a standard for ethical behaviour and quality-driven scholarly activities: excellence and accountability in every endeavour, regardless of time or place. The principle is fundamental in medical education, where the responsibilities of educators extend beyond the classroom and significantly influence patient care and public health.

The School of Medical Sciences (SMS) at Universiti Sains Malaysia (USM), has a history of over 40 years. It has achieved significant milestones in its core areas of academics, research, and professional excellence (3). To enhance its culture of excellence, it is crucial to inspire and reaffirm the *amanah* held by its academicians.

## *Amanah* for Academic Excellence

The SPICES model guides the undergraduate curriculum, which embodies six core principles: S – Student-centred, P – Problem-based, I – Integrated, C – Community-based, E – Electives, and S – Systematic (3). Initially known as SPICES 1.0, this model has been enriched and modernised to address the evolving demands of 21st-century healthcare with SPICES 2.0. This updated model incorporates new elements to prepare medical graduates for the challenges of modern healthcare systems. The model emphasises simulation-based, portfolio-based monitoring, individualised workplace learning, and competency-based education to ensure measurable outcomes and that students develop specific skills tailored to their future roles (4).

The postgraduate clinical specialist training programme at the SMS started in 1998 and has long been recognised as the cornerstone of advanced medical education in Malaysia (3). It delivers 4-year programmes (Master of Medicine, Master of Surgery, Master of Pathology, and Doctor of Public Health) designed to produce clinical specialists. With the recent amendment to the Medical Act of 1971 (Amendment Bill 2024), these programmes are now mandated to undergo accreditation by the Malaysian Qualifications Agency as a prerequisite for recognition by the Malaysian Medical Council. This development promotes rigorous quality assurance aligned with speciality-specific standards, and it reinforces the SMS’s commitment to excellence, which may eventually result in global prominence. SMS also offers research-, mixed-, and coursework-mode programmes, attracting local and international students with innovative lifelong learning.

## *Amanah* for Student Excellence

Medicine is a highly competitive field, and attracting crème de la crème students helps the SMS achieve excellence. But while attracting top-tier students is crucial, the institution emphasises the development of well-rounded, compassionate professionals who embody USM values and who are prepared for the complexities of modern healthcare. To this end, the SMS has several strategies, including mandatory professionalism assessment via the Simplified Thematic Engagement of Professionalism Scale, which evaluates professionalism within the local context (5).

Central to USM’s educational agenda is the value-driven HEBAT framework, which focuses on developing holistic, entrepreneurial, balanced, articulate, and thinking qualities. The framework is integrated into various aspects of the medical curriculum and student development programmes. USM has also initiated several programmes, including “One Student One Passport”, which is an elective exchange programme to develop intercultural competence. These programmes ensure that USM students are not only academically proficient but also well-rounded, value-driven individuals.

In addition to cultivating excellence and core values, a pivotal challenge in medical education lies in guiding postgraduate clinical students towards recognising their own cognitive limitations. This process involves navigating the eight stages of ignorance: the Age of Naivety (characterised by a lack of *amanah*), the Facts (initial knowledge acquisition), Naïveté (simplification of complexities), frustration (confronting the limits of practice and skills), expertise (a stage of confidence often fraught with overconfidence risks), Pyrrhic success (progress achieved at significant cost), ignorance (realisation of previously unrecognised gaps), and loss of insight (failure to adapt or self-reflect) (6). By addressing these concerns, SMS produces academically excellent graduates and compassionate professionals who are ethically anchored and equipped to face the complexities of modern medical practice with resilience and insight.

## *Amanah* for Research Excellence

Research clusters serve as catalysts for high-impact knowledge generation. They foster advanced innovations, enhance efficiency, and promote knowledge and experience spill overs. They offer cost-effectiveness by optimising resources and facilitating the organisation of research activities into manageable, focused groups. However, research clusters are not without limitations. They may impede their members’ ability to execute plans and can lack the flexibility required to adapt to dynamic research needs, potentially leading to inefficiencies. Addressing these challenges is essential to maximise the benefits of research clusters while mitigating their drawbacks.

Understanding the relationship between scientific productivity and research group size is crucial to optimise outcomes such as the number of publications, citation metrics, and the impact factor of the journals in which the research is published. Evidence suggests that maintaining many small research groups may increase productivity, as opposed to concentrating resources within a few large groups (7). This approach mirrors Amazon’s “two-pizza team” strategy of team sizes small enough to be fed with two pizzas. This structure encourages meaningful contributions, fosters collaboration, and promotes efficiency within research clusters while maximising individual and collective outputs (8).

The core strategy in research excellence is interdisciplinary collaboration, followed by international engagement (9). Establishing international networks and collaborations with globally recognised institutions facilitates the exchange of ideas, data, and joint publications. These benefits enhance the quality and impact of research while helping institutions thrive in a dynamic and interconnected world.

Research should not merely follow prevailing trends or adopt a bandwagon approach without strategic planning and thorough feasibility studies. Unplanned endeavours risk suboptimal outcomes while consuming substantial resources or funding. For example, the integration of artificial intelligence (AI) into medical research, although promising, is fraught with challenges. The notion “garbage in, garbage out” underscores the risks of inadequate data quality and methodological rigour in AI-driven studies (10). A cautionary parallel can be drawn from the electrification of the automobile industry, where even giant companies have incurred significant financial losses, highlighting the unforeseen complexities of market adoption. Disruption occurs in many areas, such as functional and strategic disruption, including in medical research, as we are heavily oriented towards sophisticated technology for diagnostics and treatment (11). Therefore, identifying the current and future niches is one of the key strategies for excellence.

## *Amanah* for Service Excellence

Academicians in medical schools have dual roles as educators and practising clinicians. Multitasking is essential in their roles, as it involves comprehensive academic guidance to students while also delivering outstanding clinical services to patients. Medical academicians also contribute to community health through preventive and therapeutic measures. Initiatives such as health screenings in rural and underserved areas highlight the broader influence of medical educators on public health.

Adopting a holistic approach involving collaboration with non-medical teams can improve the impact of community service efforts. By integrating expertise from social work, education, and local governance, we can address the broader determinants of health for comprehensive, sustainable outcomes. These interdisciplinary partnerships broaden the scope of outreach initiatives by aligning healthcare delivery with the community’s social, economic, and cultural needs. This approach reflects the principle of *amanah* or trust in academia, wherein medical academicians are entrusted with shaping future healthcare professionals while serving their communities. However, a significant challenge lies in capturing, organising, and showcasing related activities and their outcomes. Aligning such efforts with the Sustainable Development Goals demands innovative strategies to document and measure their impact in order to ensure their visibility and contribution to global health and development objectives.

## The Essence of Time Management

Effective time management is critical for academic achievement and professional performance, especially in medicine. Balancing the dual roles of clinical responsibilities and academic workloads requires a strategic approach beyond simple scheduling. Academicians must develop a comprehensive set of skills to steer this challenge successfully. One strategy is time-blocking, where specific periods are allocated to different tasks, including protected time for academic activities such as research and writing. This approach can enhance productivity and help maintain a structured routine, which is crucial for long-term success in medical academia. Procrastination is the most significant barrier to this balance (12). Overcoming it requires discipline, self-awareness, and practical tools to stay focused and accountable.

The concept of man hours or effective working hours can be relevant in academia, particularly for tracking productivity in research and publications. By monitoring effective hours, institutions can identify areas where mentoring or support from senior academicians through targeted interventions may be needed. Additionally, timely and efficient decision-making by top management can reduce bureaucratic delays and foster a supportive environment. Prioritising meaningful, productive working hours is essential to optimise academic output and ensure that efforts translate into impactful research and scholarly contributions.

## Conclusions

Becoming an excellent academician is no trivial undertaking. It requires a steadfast commitment to excellence in academics, student mentorship, research, and service. This multifaceted responsibility is a professional obligation and an *amanah*, a trust to be carried out with integrity and accountability. A defining trait of great achievers in academia is their mastery of time management, which enables them to balance these diverse roles. Intra- and inter-school collaboration, as well as international engagement, is the way forward for SMS.
